# COVID-19-Ratio zur aktuellen Abschätzung der intensivmedizinischen Belastungsgrenze

**DOI:** 10.1007/s10049-020-00758-9

**Published:** 2020-08-03

**Authors:** K.-G. Kanz, V. Bogner-Flatz, M Daunderer, M. Dommasch, D. Hinzmann, M. Städtler, D. Steinbrunner, Th. Weiler, K. Traunspurger, J. Buchhauser, C. Ebersperger, M. Bayeff-Filloff

**Affiliations:** 1 Regierung von Oberbayern, München, Deutschland; 2 Zentrale interdisziplinäre Notaufnahme, Klinikum rechts der Isar der Technischen Universität München, Ismaninger Str. 22, 81675 München, Deutschland; 3 Rettungszweckverband München, München, Deutschland; 4 Klinik für Unfallchirurgie, Campus Innenstadt, Klinikum der Universität München, München, Deutschland; 5 Zweckverband für Rettungsdienst und Feuerwehralarmierung Fürstenfeldbruck, Fürstenfeldbruck, Deutschland; 6 Klinik für Anästhesiologie und Intensivmedizin, Klinikum rechts der Isar der Technischen Universität München, München, Deutschland; 7 Zweckverband für Rettungsdienst und Feuerwehralarmierung Rosenheim, Rosenheim, Deutschland; 8 Zentrale Notaufnahme, RoMed Klinikum Rosenheim, Rosenheim, Deutschland; 9 Geschäftsführung, Starnberger Kliniken GmbH, Starnberg, Deutschland; 10 Bayerisches Staatsministerium des Inneren, für Sport und Integration, München, Deutschland

Aufgrund der Coronaviruspandemie hatte die Bayerische Staatsregierung unter Führung von Ministerpräsident Dr. Markus Söder am 16.03.2020 den Katastrophenfall für Bayern ausgerufen [1]. Um die Krankenhäuser auf die zu erwartenden massiven Fallzahlsteigerungen vorzubereiten, war angesichts besonders schwerer und lebensbedrohlicher Krankheitsverläufe und Erfahrungen mit erheblichen Engpässen in anderen Staaten zum Schutz der Bevölkerung eine möglichst umfangreiche Ausweitung der zur Verfügung stehenden Behandlungskapazitäten für die Versorgung von COVID-19-Patienten erforderlich; daher verpflichtete das Bayerische Staatsministerium für Gesundheit und Pflege die Krankenhäuser durch Allgemeinverfügung vom 19.03.2020, soweit medizinisch vertretbar, bis auf Weiteres alle planbaren Behandlungen zurückzustellen oder zu unterbrechen [2]. Für die Organisation der Krankenhausbelegung wurde mit einer weiteren Allgemeinverfügung am 24.03.2020 in jedem der 26 bayerischen Zweckverbände für Rettungsdienst und Feuerwehralarmierung (ZRF) die Funktion eines Ärztlichen Leiters Führungsgruppe Katastrophenschutz (Ärztlicher Leiter FüGK) eingerichtet und bayernweit ein einheitliches, IT-gestütztes System zur Erfassung der Behandlungskapazitäten und COVID-19-Patienten eingeführt [3, 4]. Die Fallzahlen und Belegungsdaten waren auf Grundlage des IT-Programms IVENA Sonderlage verbindlich und fortlaufend über einen Internetzugang zu erfassen [6]. Darüber hinaus wurden die Krankenhäuser verpflichtet mit allen geeigneten Mitteln Kapazitäten zur Behandlung von COVID-19-Patienten oder zur Entlastung anderer Krankenhäuser, die vorrangig für COVID-19-Patienten herangezogen werden, auszubauen [3, 4, 7].

Im Rahmen der ersten Welle der Coronaviruspandemie in Deutschland war der Regierungsbezirk Oberbayern am stärksten betroffen, es waren bis zu 345 Beatmungsplätze mit COVID-19-Patienten belegt, im Rettungsdienstbereich (RDB) München bis zu 166, im RDB Rosenheim bis zu 58 und im RDB Fürstenfeldbruck bis zu 36 (Tab. 1).

**Table 1:** 

	Datum	Ist-ICU-Betten	Max.-ICU-Betten	Non-COVID-19	COVID-19	Freie Max.-ICU-Betten	COVID-19-Ratio
Regierungsbezirk Oberbayern	06.04.2020	1046	1599	471	345	388	1,0
Rettungsdienstbereich München	06.04.2020	543	883	279	166	126	0,8
Rettungsdienstbereich Rosenheim	10.04.2020	109	158	44	58	42	0,7
Rettungsdienstbereich Fürstenfeldbruck	11.04.2020	83	91	23	36	24	0,7

*ICU* „intensive care unit“

Die Abschätzung der „surge capacity“ in Bezug auf die benötigten Beatmungsplätze ist, neben der Zahl der intensivpflichtigen COVID-19-Fälle, ein wesentlicher Faktor für die Beurteilung der aktuellen Lage. *„Der Begriff ‚Surge Capacity‘ beschreibt die Fähigkeit eines Gesundheitssystems, plötzlich seine Möglichkeiten und Betriebsführung über das normale Maß hinaus zu erweitern, um den gestiegenen Bedarf an medizinischem Personal und an Diensten während eines Großschadensereignisses zu bewältigen. Der englische Begriff ‚surge‘ wird vom Wörterbuch Merriam-Webster als ein plötzliches Anwachsen auf außergewöhnliche oder abnormale Werte definiert“* [5]. In der Logistik wird der Begriff im Übrigen als Belastungsgrenze bezeichnet.

In der akuten COVID-19-Pandemielage haben wir für die Abschätzung der intensivmedizinischen Belastungsgrenze eine sogenannte COVID-19-Ratio, das Verhältnis freier ICU(„intensive care unit“)-Betten zu mit COVID-19 belegten ICU-Betten, entwickelt und eingesetzt. Wenn beispielsweise 2 intensivpflichtige Patienten akut aufgenommen werden müssen und noch 1 weiteres freies ICU-Bett zur Verfügung steht, beträgt diese 0,5. Wenn 4 freie ICU-Betten vorhanden sind, 2,0. Bei gleichbleibender Zahl an freien ICU-Betten wird bei einer Zunahme der COVID-19-Fälle die Ratio kleiner und größer bei einer Abnahme der COVID-19-Fälle. Analog wird bei gleichbleibender Zahl der COVID-19-Patienten bei einer Zunahme der freien ICU-Betten die Ratio größer und kleiner bei einer Abnahme der freien ICU-Betten. Die COVID-19-Ratio ist somit eine einfach zu berechnende operationale Kenngröße für Einsatzleitungen bzw. Notaufnahmen oder Intensivstationen.

Der Verlauf der Belegung der ICU-Betten sowie die hierzu korrespondierende COVID-19-Ratio sind exemplarisch für den Zeitraum vom 1. April bis 31. Mai 2020 für den RDB München und den RDB Rosenheim dargestellt. Im RDB München veränderte sich bereits Mitte April die COVID-19-Ratio nach einer wesentlichen Aufstockung der ICU-Betten von 1,0 auf 2,0 (Abb. 1a). Im RDB Rosenheim lag im April die COVID-19-Ratio bei Werten um 1,0 und verbesserte sich erst Mitte Mai bei einem gleichzeitigen Rückgang der COVID-19-Fälle und Zunahme der freien ICU-Betten auf Werte über 2,0 (Abb. 1b).

**Figure 1:**
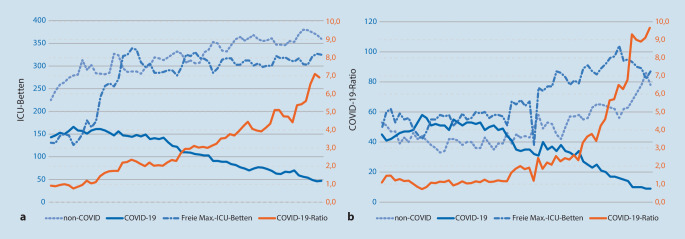

Die COVID-19-Ratio ermöglicht Einsatzleitungen, Notaufnahmen und Intensivstationen eine einfache Abschätzung der oben angeführten „surge capacity“ im Hinblick auf die Reservekapazität von Beatmungsplätzen. Nach unseren Erfahrungen weisen Werte unter 1,0 auf eine kritische COVID-19-Sonderlage hin und Werte zwischen 1,0 und 2,0 auf eine angespannte Situation. Inwieweit die COVID-19-Ratio als prognostischer statistischer oder epidemiologischer Parameter für das Infektionsgeschehen sinnvoll ist, können wir nicht beurteilen [8]. Es bietet sich an, die COVID-19-Ratio prospektiv zu evaluieren, sowohl in Bezug auf die von uns subjektiv vorgeschlagenen Grenzwerte als auch im Hinblick auf eine mögliche prognostische Risikoeinschätzung der infektiologischen Gefährdungslage.

## References

[1] Bayerische Staatskanzlei (2020) Corona-Pandemie / Bayern ruft den Katastrophenfall aus / Veranstaltungsverbote und Betriebsuntersagungen. https://www.bayern.de/corona-pandemie-bayern-ruft-den-katastrophenfall-aus-veranstaltungsverbote-und-betriebsuntersagungen/. Zugegriffen: 16. Juni 2020

[2] Bayerische Staatskanzlei (2020) Veröffentlichung BayMBl. 2020 Nr. 151 vom 25.03.2020. https://www.verkuendung-bayern.de/baymbl/2020-151/. Zugegriffen: 16. Juni 2020

[3] Bayerische Staatskanzlei (2020) Veröffentlichung BayMBl. 2020 Nr. 164 vom 01.04.2020. https://www.verkuendung-bayern.de/baymbl/2020-164/. Zugegriffen: 16. Juni 2020

[4] Bayerische Staatskanzlei (2020) Veröffentlichung BayMBl. 2020 Nr. 253 vom 11.05.2020. https://www.verkuendung-bayern.de/baymbl/2020-253/. Zugegriffen: 16. Juni 2020

[5] Bey T, Koenig KL, Barbisch DF (2007). Das Konzept von „Surge Capacity“ im Katastrophenfall. Notfall Rettungsmed.

[6] Flemming A, Hoeper MM, Welte T, Roesler P, Ringe B (2020). Rettungsdienst: Schneller in die richtige Klinik. Dtsch Arztebl.

[7] Vetzke MM, Kemming GI (2019). Der Aufwachraum als Behandlungsplatz – Vorbereitet im Falle von Terror vor den Toren kommunaler Kliniken. Notfall Rettungsmed.

[8] Weiß C (2018). Der Schluss von der Stichprobe auf die Grundgesamtheit. Notfall Rettungsmed.

